# Assessing health systems in low-resource settings: some conceptual and methodological dilemmas

**DOI:** 10.1186/1472-6963-14-S2-O24

**Published:** 2014-07-07

**Authors:** Dina Balabanova

**Affiliations:** 1 London School of Hygiene and Tropical Medicine, UK

## 

Demands for health system assessments have increased in recent years, partly due to a gradual shift in funding patterns – from investment in vertical disease-focused programmes, to horizontal systems aiming to strengthen health systems as a whole – and due to a need to establish the impact of this investment. There is also a pressure to monitor progress related to Millennium Development Goals (MDG) or other internationally agreed developmental targets in order to inform ongoing political processes. Citizens, health services users and communities are increasingly empowered to hold the health systems to account. Yet there are huge challenges in assessing health systems in middle and low-income settings due to scarce or poor quality data, and a lack of capacity to collect, analyze and use data.

This presentation will explore some of the methodological and practical challenges faced in seeking to analyze health system performance, particularly in low-resources settings. A range of approaches for health system assessment that are commonly used will be discussed: from toolkits seeking to obtain standardized information on a range of indicators, to more exploratory rapid appraisals and theory-based evaluations. Conceptually, there are still debates about how to measure impact, how to undertake multi-method evaluations, and how to ensure that research findings inform practice. The talk will discuss the shift from a more traditional focus on performance assessment to a growing interest in interpretative health systems and policy research.

